# Data-Driven Design Rules for Three-Dimensional Photonic Crystals

**DOI:** 10.1021/acsomega.6c00256

**Published:** 2026-03-10

**Authors:** Rose K. Cersonsky, Saswat K. Nayak, Seungmin H. Lee

**Affiliations:** † Department of Chemical and Biological Engineering, 6111 University of Wisconsin - Madison, Madison, Wisconsin 53706, United States; ‡ Department of Materials Science and Engineering, University of Wisconsin - Madison, Madison, Wisconsin 53706, United States; § Data Science Institute, University of Wisconsin - Madison, Madison, Wisconsin 53706, United States; ∥ Department of Chemical and Biological Engineering, Notre Dame University, South Bend, Indiana 46556, United States

## Abstract

Photonic crystals are crystalline systems composed of multiple materials whose patterning results in the selective reflectance of light. Historically, design principles for three-dimensional photonic crystals have remained limited to how to *optimize* photonics response, thereby limiting synthetic guidance in non-ideal systems. This work introduces a data-driven approach to uncover such principles; we transform a data set comprising 1,200 crystalline templates (and tens to hundreds of band structures per template) from band structures into photonic densities of states (PDOS), which serve as statistical fingerprints for property-structure analyses. We exploit hybrid supervised–unsupervised dimensionality reduction and clustering to reveal low-dimensional maps of this high-dimensional latent space that capture both structural similarity and gap size, enabling sensitivity analyses across symmetry classes and material distributions. Results show that photonic band gap (PBG) size is primarily correlated with the volume fraction and connectivity of high-dielectric media (optimal gaps occur at fractions of 0.2-0.3), while global lattice symmetry plays a secondary, less critical role. Networks with tetrahedral or gyroidal connectivity consistently support photonic band gaps even under local and global symmetry distortions. These findings broaden conventional design rules to include aspects of local topology and material complexity, providing a foundation for the future design of photonic structures.

## Graphical abstract

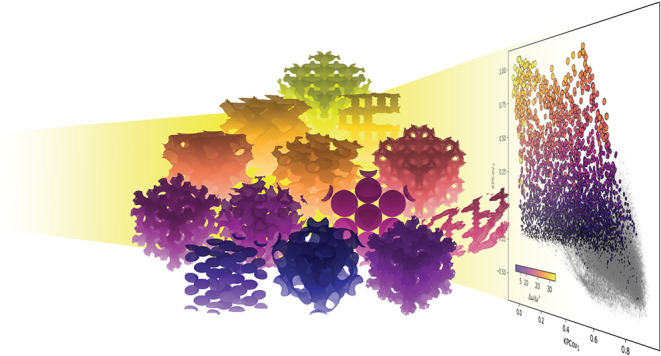

## 1 Introduction

Color in materials originates from diverse physical mechanisms ranging from electronic transitions to structural interference. Among these, photonic crystals, periodic arrangements of dielectric media, stand out for their ability to manipulate light through crystallographic geometry. By introducing mesoscopic patterning, these structures create photonic band gaps (PBGs),^1–3^ frequency ranges where light propagation is forbidden, enabling applications in optical communication, sensing, and energy harvesting.

The optical behavior of such structures is captured in their photonic band structure, determined by the spatial variation of the dielectric function, *ε*(**r**). The transmittable frequencies ω result from the eigenproblem

1 $$\nabla \times \frac{1}{\varepsilon \left(r\right)}\nabla \times H={\left(\frac{\omega }{c}\right)}^{2}H$$

where *c* is the speed of light and *a* is the characteristic length scale of the periodic pattern. The eigenvalues are naturally expressed in units of *c*/*a*, where *a* is the lattice parameter.

So, what makes a three-dimensional pattern of *ε*(**r**) result in a band gap? What are the design principles that govern this interesting phenomenon? Early work sought to limit the design space through symmetry and physical reasoning, yielding guidelines for large band gap formation in face-centered cubic lattices^1,4–7^ and connected dielectric networks.^8–11^ Yet, emphasizing only high-gap configurations has led to structures that are challenging to fabricate.^12–20^ Thus, to advance design, we must instead identify the broader structural principles that allow a band gap to emerge at all, not merely those that maximize it.

Current modern tools of machine learning (ML) offer great promise in identifying data-driven design principles. Typically, such a workflow identifies characteristics or representations of a crystal structure and builds predictive models for a set of properties. In this context, this would constitute inputting *ε*(**r**) into an ML pipeline to predict ω, and then using explanatory tools to determine the sensitivity of ω to small changes in *ε*(**r**). Thus, these studies first require the ability to encode crystallographic patterns *ε*(**r**) whose mathematical comparison is a reasonable proxy for similarities in the modes in ω. However, the choice in mathematical representation provides a considerable challenge, both conceptually and practically. Consider scaling the dielectric function by a factor *s*, such that **r**′ = *s* **r** and ∇′ = ∇/*s*. Substituting yields

2 $$\nabla^{\prime }\times \left(\frac{1}{\varepsilon \left(r^{\prime }/s\right)}\nabla^{\prime }\times H\left(r^{\prime }/s\right)\right)={\left(\frac{\omega }{sc}\right)}^{2}H\left(r^{\prime }/s\right)$$

showing that the eigenfrequencies scale as ω′ = ω/*s*. Thus, the solution at one length scale determines those at all others.

This poses a natural problem in applying descriptors from the materials informatics community, who typically ground their representations, even those that are scale-equivariant^23^ or symmetrized^24^, on atoms as graph loci or points from which to define distance matrices. For metamaterials consisting of material across space, this construction is no longer appropriate or representative. Additionally, an atom-centered approach limits analysis to dielectric patterns, where the high-dielectric medium sits on the lattice sites, despite the multitude of so-called “inverse” photonic crystals possible.

One could consider representing *ε*(**r**) based on scale-covariant operations on three-dimensional voxel images, as is common in image-processing pipelines.^25^ However, traditional topological measures adopted from point cloud and image analyses, while effective in long-range, amorphous, or quasiperiodic systems^26,27^ fail to capture meaningful variation in strictly periodic lattices where topology is invariant under symmetry operations^28^ and are difficult to meaningfully normalize across systems with different voxel densities.^29^ Thus, any topological analyses or geometric learning are limited to crystals within the same periodic symmetry or consistent densities of voxels, as has been seen in the deep learning analyses of photonic structures.^30^

Finally, the exploitation of Fourier-based analyses for materials informatics is still relatively underdeveloped.^31,32^ The computation of eq 1 itself involves the repeated application of curl operators and spatial convolutions with the inverse dielectric function, operations that are most efficiently performed in reciprocal space by using fast Fourier transforms (FFTs). In practical implementations such as MIT Photonic Bands (MPB),^2^ this entails iterative evaluations of the Maxwell operator via forward and inverse FFTs, enabling efficient solution of the eigenvalue problem for complex, spatially varying permittivity profiles. Despite the algorithmic efficiency of FFT-based solvers, the numerical treatment of low-symmetry systems, particularly triclinic or aperiodic geometries, remains computationally demanding, and thus characterizing *ε*(**r**)­(*r*) via FFTs for data-driven studies is no more trivial than computing ω.

In the absence or failure of mathematical means to compare diverse sets of *ε*(**r**), it is not trivial to build structure–property relationships through machine-learning analyses. However, consider the overall objective—to identify trends in photonic properties as a function of crystallographic symmetry. Within this task, there is still considerable knowledge to be learned from the inverse: property-structure relationships. In other words, if one compares the property space of three-dimensional photonic crystals, what can be learned about the rules of structural design?

By examining the similarity in the photonic densities of states (PDOS) as a proxy for similarity in *ε*(**r**), we can instead treat the PDOS as a statistical fingerprint of how geometry and crystallography shape optical response. In this work, both unsupervised and hybrid supervised–unsupervised approaches are used to identify clusters and classes within the DOS landscape, followed by an analysis of the crystallographic motifs that define each group. This framework enables the connection of photonic behavior to underlying structure without the drawbacks of incomplete or scale-dependent geometric descriptors.

## 2 Methods and Results

The data set published in Cersonsky et al.^33^ contains roughly 1,200 crystalline “templates” and their photonic band structures computed across varying radius *r* of dielectric spheres on each lattice site and the ratio of high and low dielectric media ϵ. The original data set contains the first 20 bands across the irreducible Brillouin zone at ϵ ∈ [2,16] (and their reciprocals); here, the analysis is limited to ϵ = 16 (and its reciprocal), an extreme corresponding to loss-less silicon occupying the dielectric spheres or background material. This simplifies the discussion solely to that of structure and symmetry, although similar analyses may be performed to understand the interplay with ϵ. While the majority of crystals demonstrate monotonic gap size with *ε*,^34^ several crystals exhibit a nonmonotonic relationship between *ε* and gap size,^33^ and thus small gap sizes at this *ε* do not preclude larger gaps at lower *ε*. A schematic of this data space is shown in Figure 1(left).

**Figure 1:**
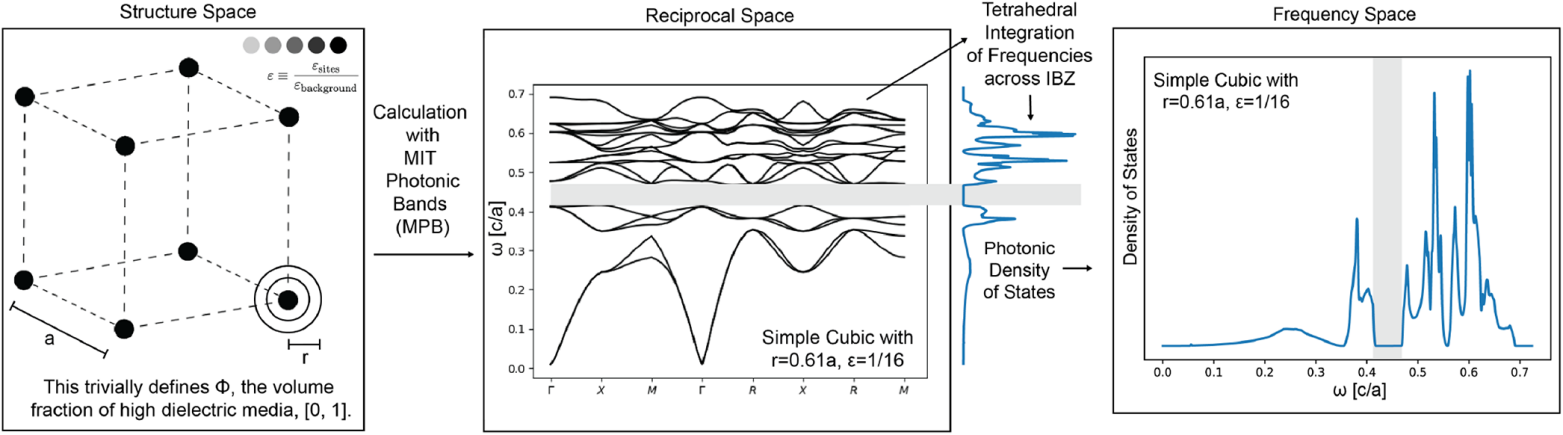
Procedure for data curation and production for this text. (left) Structures were queried at different radii of a dielectric sphere, and (center) the first 20 photonic bands were computed with MIT Photonic Bands.^2^ (right) From these band structures, the photonic density of states (PDOS), which represents the number of mode solutions at any given frequency, was computed using the tetrahedral integration method formalized in ref 21 and implemented by ref 22. This yields a summary of the band structure for the first 20 bands across a unifying length scale in frequency space, as opposed to the center panel, which is computed for *k*-vectors within reciprocal space.

A photonic band spectrum is typically represented in reciprocal space and scaled frequencies; this sort of data object is nonstandardized and cannot be compared readily across different symmetry classes or lattice parameters. These spectra are thus transformed into the photonic density of states (PDOS), a standardized data object expressed in consistent units and comparable across multiple systems. A density of states is formally defined as

3 $$\mathrm{PDOS}\left(\omega \right)=V_{BZ}^{-1}\underset{\mathrm{bands}\;n}{\sum }{\;\int }_{BZ}\;\delta \left(\omega -\omega_{n}\left(k\right)\right)d^{3}k$$

The density of states can be determined by taking a weighted sum of frequencies within the irreducible Brillouin zone (IBZ) in an established procedure known as “tetrahedral integration”.^21^ We adopted the open-source implementation of Liu et al.^35^ available on GitHub.^22^ This algorithm partitions the Brillouin zone into tetrahedra defined by frequency data at discrete k-points and analytically integrates the PDOS contributions within each volume element. Parameters such as the k-point mesh (20,000 bins), frequency limits, and histogram resolution were standardized across all structures to ensure consistent numerical treatment.

For each crystal structure, radius, and dielectric contrast, the original data set contained a set of *k*-point vectors and the corresponding frequencies for the first 20 photonic bands. The *k*-point vectors spanned the IBZ and included 10 interpolants between *k*-points. To enable the determination of frequencies for arbitrary points within the IBZ, we fit each band to an independent smooth radial basis interpolant using scipy,^36^ and reevaluated the frequencies across a uniform 10 × 10 × 10 Monkhorst–Pack grid in reciprocal space, with reciprocal vectors determined by spglib.^37^ This procedure enabled us to leverage existing data; we validated this procedure by choosing several arbitrary IBZ points and comparing interpolated versus calculated frequencies.

For each computed band structure, this procedure yields a 20,000-point vector representing the photonic density of states over all relevant frequencies, denoted as PDOS (ω) and illustrated in Figure 1 (right). Because the eigenfrequencies of the 20 photonic bands scale with the characteristic lattice length (eq 2), the frequency bins are normalized by min (ω_20_); the resulting scaled quantity is simply referred to as PDOS.

Following literature on the electronic densities of state,^38^ and to smooth this PDOS, the PDOS is then projected onto principal components, as determined by a subset of 1000 spectra selected by furthest point sampling.^39,40^ This is expressed mathematically as

4 $$X\approx \mathrm{PDOS}\;\cdot {\;W}^{T}$$

where *W* are the transpose of the principal components and *X* is the projection of the PDOS along these PCs. For a sufficiently large number of PCs, it is then trivial to reconstruct the PDOS as

5 PDOS=XW

In this case, this leads to the results of Figure S3: the PCA projection retains 99.9% of the variance in the 20,000-length PDOS vectors with only 130 components and steadily reduces the mean absolute error with further components. Furthermore, considering the secondary task of correctly identifying zero-mode frequencies (frequencies within the band gap), this is possible with as few as 60 PC components.

However, this approach leads to misleading results as to which crystals exhibit similar PDOS’s. Because high-mode frequencies dominate distance calculations, PDOS’s with similar magnitudes yet dissimilar shapes obtain high similarity scores. As shown in Figure 2, for the PDOS of *cF*8 – *C* at *r* = 0.33 and *ε* = 16 (blue), using this approach leads to *oP*280 – *C* at *r* = 0.0869 and *ε* = 16 (green) being registered as the structure with the most similar PDOS. However, it is typical to want to emphasize similarity in the PDOS shape and the zero-mode frequencies, where PDOS = 0. Thus,

6 $$X^{\prime }\approx \exp^{-\mathrm{PDOS}}W^{\prime }$$

This modification will more heavily weight similarity in zero-mode frequencies (band gaps) rather than high-mode frequencies, and results in *cF*64 – *FeF*_3_ at *r* = 0.215 and *ε* = 16 (orange) being identified as the structure with the most similar PDOS to *cF*8 – *C* in Figure 2. *X*′ is projected in Figure 3a and an analogous figure for *X* is included in Figure S3.

**Figure 2:**
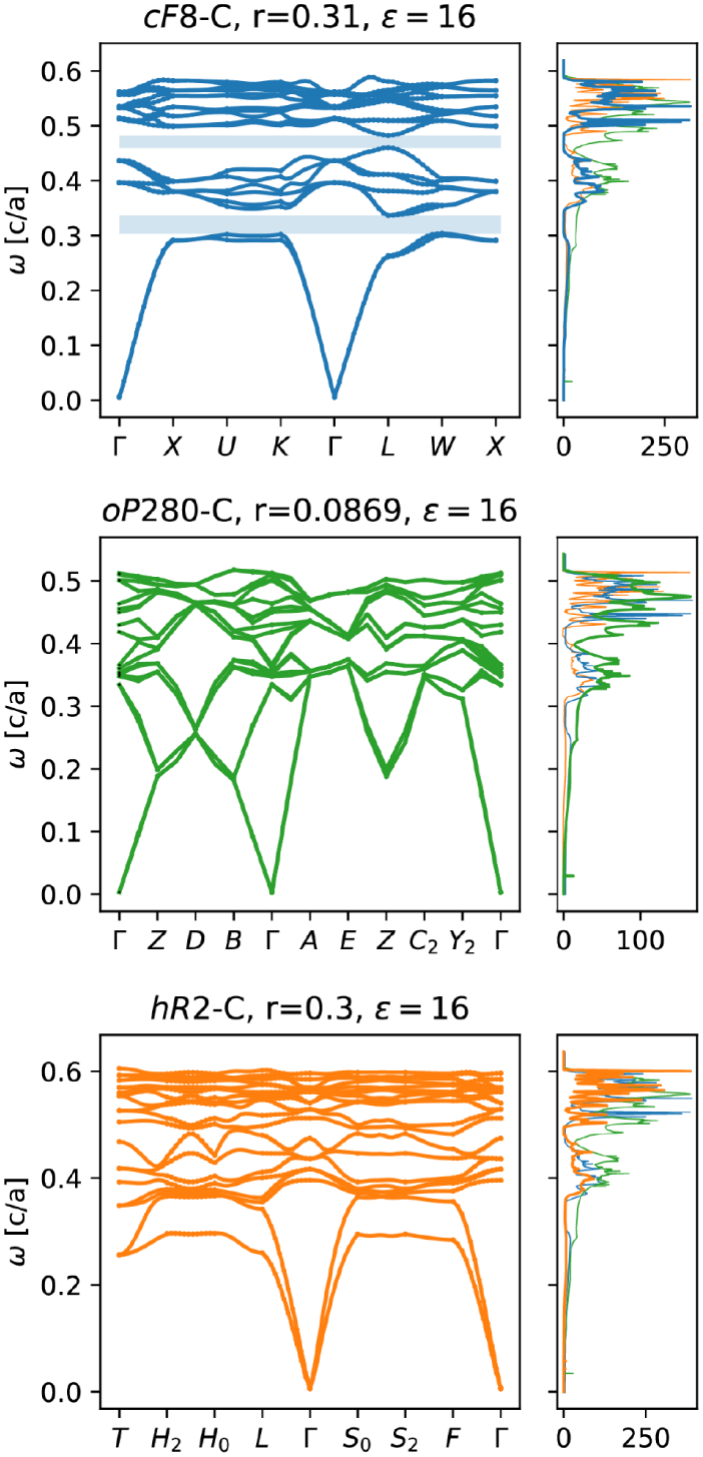
Nuance in comparing densities of states using Euclidean distance (as is done in standard PCA) for the archetypal diamond structure (blue, *cF*8 – *C* at *r* = 0.31 and *ε* = 16). A standard PCA will yield high similarity scores for *cF*8 – *C* at *r* = 0.33 and *ε* = 16 and *oP*280 – *C* at *r* = 0.0869 and *ε* = 16 (green), whereas a PCA on the negative exponent of the PDOS will yield high similarity scores for *cF*8 – *C* and *hR*2 – *C* at *r* = 0.3 and 16 (orange).

**Figure 3:**
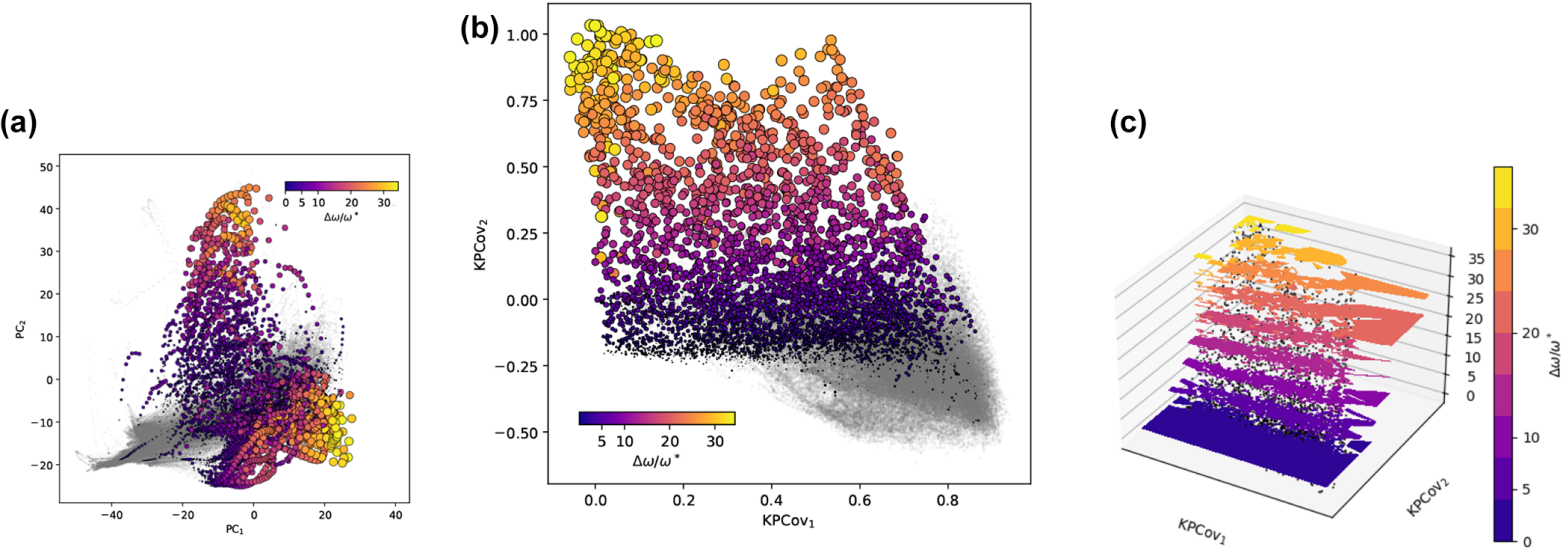
Unsupervised and hybrid supervised–unsupervised mappings of the photonic densities of the state. Pricipal components analysis of the photonic densities of state, highlighting similarities in zero-mode frequencies by eq. 6. (b) Kernel principal covariates analysis of (a), highlighting similarities in zero-mode frequencies and rotating the latent space to correlate with gap size. (c) Contour plot on interpolated grid data for b. Scatterpoints denote original data.

Herein lies an intermediate goal: a map (Figure 3a) in which the similarity of PDOS for specific crystal structures at different *r*. This map, while visually appealing, only truly delineates the two classes of large photonic band gap crystals, which, in line with earlier work,^4,6,8^ are strongly related to the diamond lattice with connected tetrahedral networks. The lower population generally consists of high-symmetry (face-centered cubic and body-centered tetragonal) lattices with large band gaps at low band numbers and frequencies, whereas the upper population comprises lower-symmetry lattices whose large band gap is shifted higher in band number due to band folding and superposition.^41^ However, this shift does not necessarily coincide with a frequency shift, as seen by the lack of correlation between the first covariate and the midgap frequencies (Pearson correlation coefficient of −0.17).

In order to better highlight the intermediate and small band gap structures, we utilize Kernel Principal Covariates Analysis (KPCovR,^40,42^ ). Kernel Principal Covariates Analysis constructs a low-dimensional map that encodes both similarity in mathematical features (here, *X*′) and some learnable property (here, gap size). To first test the learnability of gap size from *X*′, we perform a regularized kernel regression, as shown in Figure S2. The KRR is fit (using scikit-learn, an RBF kernel with default Gaussian width, and a regularization strength of 0.3) on a subset of 90% of the data set exhibiting photonic band gaps, roughly 4,000 data points, and demonstrates performance on the remaining 400 data points. As shown in Figure S2, this procedure results in *R* ^2^ = 0.78 on the out-of-sample data, which is sufficient for incorporating collinearities into a KPCovR map.^42^

The KPCovR map is then constructed using scikit-matter,^40^ a radial basis kernel with default kernel width, and a mixing parameter of 0.9 (this parameter encodes how much to retain KPCA-like variance versus alignment with the property of interest; α = 0.9 more heavily weights the former). The KPCovR procedure uses the same regularized kernel regression as in Figure S2 and the training set configurations. The maps resulting from different values of α are shown in Figure S6. The benefit of such a map is to provide spatial ordering of data points as it relates to the similarity in representation and in property space. Thus, we can analyze the classes of photonic crystals for different sizes of PBG. As shown in Figure 3b, this effectively “unrolls” the latent space from Figure 3a.

Given the correlations of this latent space with the band gap size, sensitivity analyses of the latent space can serve as indicators of gap size sensitivity. Figure 3c demonstrates the general relationship between the latent space dimensions and the gap size, using an even interpolant grid (using scipy.interpolate.grid_data on the first two latent dimensions) and contouring the levels within the data. This demonstrates a higher sensitivity within the second dimension than the first—in other words, the structural changes that correlate with the first dimension have less impact than those along the second dimension.

Using chemiscope.org,^44^ we can observe trends in symmetry class and connectivity along the first and second latent dimensions, which we quantify through kernel density estimation. For several characteristics, we computed the relative density difference across the latent dimensions. Figure 4a shows the relative density estimations for different periodic box types across the first latent dimension. The relative KDE *R*_c_(*x*) is defined as

7 $$R_{c}\left(x\right)=\frac{{\mathrm{p̂}}_{c}\left(x\right)-\mathrm{p̂}\left(x\right)}{\mathrm{p̂}\left(x\right)}$$

where *p*(*x*) is the density of points at coordinate *x*, and the subscript in *p*_*c*_ denotes the density of points within category *c* at coordinate *x*. This analysis shows a greater number of cubic structures on the left side of the plot, with an increase in lattice diversity as we move right. We have omitted the curve corresponding to triclinic lattices (“a”), as it corresponds to a much smaller set of data points (4̃0), and its relative population is not statistically representative. This analysis shows that this first dimension largely correlates with the degree of symmetry in the crystal lattice, where cubic lattices are considered the highest symmetry.

**Figure 4:**
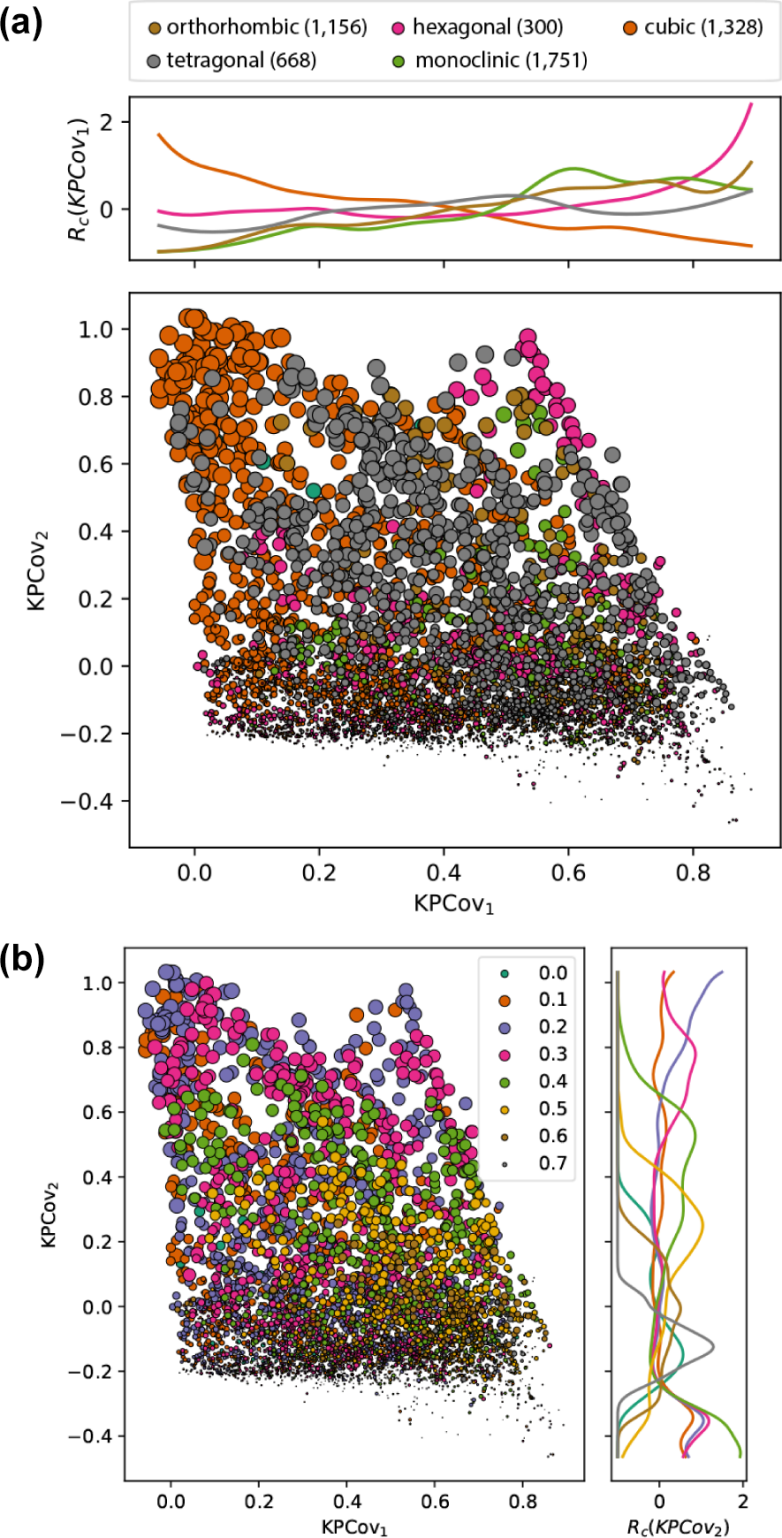
Relative kernel density estimations of the box lattice (top) and volume fraction of the high dielectric medium (bottom) across the KPCovR space. Relative KDE of different crystallographic lattices across the first latent space dimension. The legend denotes the Pearson symbol for each box type (and number of relevant data points), where *c* is cubic, *h* hexagonal, *m* monoclinic, *o* orthorhombic, and *t* tetragonal. (b) Relative KDE of different volume fractions across the second latent space dimensions.

The second dimension, qualitatively, resembles the volume fraction of high-dielectric medium in the system—this is confirmed with quantitative analyses, which show that band gaps peak at volume fractions of 0.2–0.3, with lower and higher volume fractions peaking at lower values of the second latent dimension. These sensitivity analyses confirm that gap size is most largely affected by the fraction of high-dielectric media and that symmetry class will not prohibit gaps from forming, but the largest band gaps will only exist within high-symmetry systems.

We can further parse this latent space by performing clustering analysis to determine regions of similar PDOS and gap properties. Given the relatively uneven distribution of data points in the map, hierarchical or density-based clustering algorithms are most well-suited,^45^ and thus we perform Agglomerative Clustering using scikit-learn on the first two components of the KPCovR map for the PDOSs exhibiting band gaps, using a distance threshold of 4 (this parameter is chosen based on the convergence of similarities within each cluster). The interested reader can refer to the Supporting Information containing interactive visualization of this data set for further exploration via the online tool chemiscope.org.^44^

In the upper left of Figure 5, the green cluster represents structures currently well understood by the larger community:structures forming tetrahedral or gyroidal networks. Of note is that, while many of these structures sit within the face-centered cubic lattice group, there are examples of body-centered and primitive tetragonal lattices. This confirms that it is possible (albeit less statistically likely) to obtain large band gaps in noncubic systems, provided we retain interconnected networks of high- and low-dielectric media. This has also been suggested by literature.^13,46^ These systems all have low-frequency gaps with few high-mode states below the band gap (as exemplified by the low peaks prior to the band gaps).

**Figure 5:**
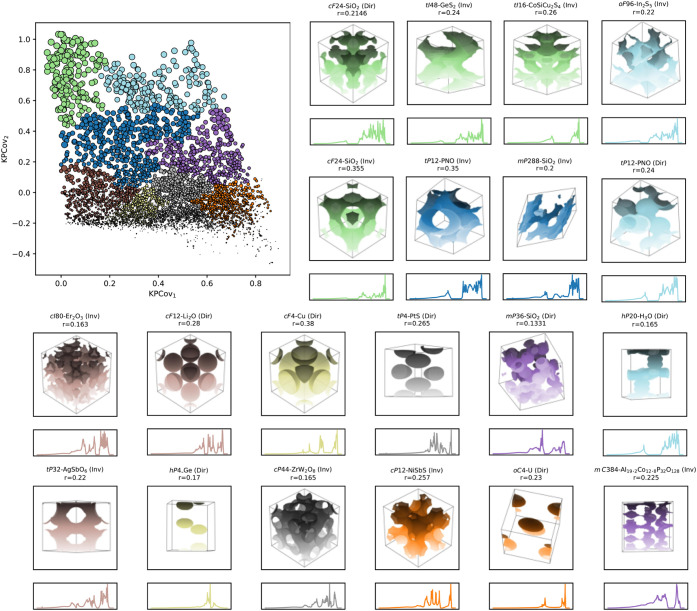
Clustering analysis on the KPCovR latent space, with insets corresponding to representative examples from each class. Insets are colored corresponding to the analogous cluster. Isosurfaces were generated by voxel fields generated by the freud software.^43^

Generally, moving downward in the map coincides with a shift in the zero-mode states to higher frequencies, with a larger number of high-density modes at varied low frequencies. This appears to result from the reduction in the volume fraction of the high-dielectric material of otherwise PhC crystals. It also appears that the roughness of the high-dielectric surface increases with this dimension:in systems where the constituent spheres merge to form smooth, continuous surfaces, the photonic band gap is higher, whereas when their overlaps result in bumpy surfaces or sharp discontinuities, this directly impacts the band gap size. These modifications introduce multiple high-mode peaks into the low-frequency regime; this is corroborated by the increase in multigap structures in the lower left of the latent space, as shown in Figure S4. This aligns with the understanding of defect modes being introduced into the band structure with rough or defected systems, as has been suggested by the literature.^47^ As we move rightward on the map, this generally coincides with the smooth growth of the initial peak in the PDOS; this appears to result from the symmetry reduction of topological networks, where the networks resemble those further left but with small perturbations or lower crystalline symmetry.

This analysis demonstrates how photonic band gap formation in three-dimensional photonic crystals is governed by a nuanced interplay among lattice symmetry, network connectivity, and material distribution. While high-symmetry lattices such as face-centered cubic remain optimal for achieving large gaps, these findings reveal that gap size is more sensitive to the volume fraction and surface complexity of the high-dielectric medium than to global symmetry distortions. Kernel-based mapping and clustering highlight that interconnected networks of dielectric media, particularly those resembling tetrahedral or gyroidal motifs, are ideal even when the global or local symmetry is distorted or modified. These insights broaden traditional design principles by emphasizing local structural features and material topology over symmetry alone, offering a pathway for rational design strategies in both ordered and complex photonic architectures.

## 3 Discussion

This study has presented a new lens for property-structure analysis of the data set published in Cersonsky et al.,^33^ aimed at identifying design principles as they affect photonic band gap structures. This analysis confirms previous observations in the literature focused on the *maximization* of photonic band gaps, namely, that they are most optimized in face-centered cubic lattices containing tetrahedral or gyroidal junction symmetry. However, the analysis adds several new layers of nuance to these design principles, several of which are open areas for exploration and quantification.

First, photonic band gaps are more sensitive to the density of high-dielectric material in the system than to general distortions of lattice symmetry, with photonic band gaps most likely to occur when the volume fraction of high-dielectric medium is 0.2–0.3, and gap size dropping quickly as the volume fraction rises or falls. This is demonstrated by the sensitivity analyses shown in Figure 3c. Similarly, qualitative analyses suggest that gap sizes are greatly affected by the roughness of the high-dielectric surface and the complexity of the networks. These observations imply that in particle-assembled systems (such as those formed via DNA-mediated colloidal crystallization,^19,48^ depletion interactions,^49^ or entropy-driven assembly^13,15,50,51^ ) precisely controlling the particle interaction profile is often a more decisive design variable than achieving perfect global symmetry. Unlike top–down lithographic or layer-by-layer techniques, these techniques rely on an emergent structure. The resulting photonic quality depends innately on whether the interaction potential enforces the correct local coordination environment and connectivity, which determine how high-dielectric networks percolate. Experimental work on colloidal diamond and tetrahedral networks has shown that small modifications to the interaction potential (e.g., patch geometry, DNA linker valence, and softness of the repulsive core) control whether the assembled structure preserves the required local tetrahedral symmetry and maintains the dielectric-fraction distribution needed to support a full band gap.^16,20,49,52^

A qualitative observation is that photonic band gaps are often found in systems where the high- and low-dielectric media adopt similar topologies at a volume fraction of 0.2–0.3. While this has been suggested in the literature,^53,54^ it remains an open challenge to rigorously and numerically validate this observation, particularly given the shortcomings of topological analyses for periodic or binary systems.

## 4 Conclusions

The results of this analysis are not intended to be conclusive on the subject, but rather to add a layer of data-driven analyses to the pursuit of a larger open question. Interested readers are encouraged to engage with and analyze the open repository of data available at DOI 10.5281/zenodo.17536659. From a machine-learning perspective, the advent of geometric learning for predicting tensorial properties holds particular promise and could provide surrogate modeling for full structural analyses. Furthermore, such mappings could, in principle, guide the inverse design of photonic waveguides by identifying regions of the photonics maps that are accessible through different fabrication techniques or material platforms, thereby enabling more targeted and efficient design optimization. Thus, we hope that this data repository can serve as a launching pad for further inquiry and science.

## Supplementary Material



## Data Availability

The data sets analyzed in this study and the corresponding code are available in the Zenodo repository at DOI: 10.5281/zenodo.17536659.

## Notes

Contains additional supporting figures to the text and discussion of negative results for different structure–property mapping procedures, namely: Figure S1: information loss in reducing photonic densities of state (PDOS) to their principal components. The black line denotes the mean absolute error (MAE) in PDOS construction across all structures. The red line denotes the mean accuracy across all structures in identifying zero-mode frequencies. Shaded regions denote one standard deviation. Figure S2: regularized kernel ridge regression relating PDOS to gap size. Figure S3: principal components analysis of the photonic densities of state, highlighting similarities in high-mode frequencies. Figure S4: Figure 3b, colored by number of band gaps. Figure S5: Figure 3b, colored by Bravais lattice. Figure S6: effect of rotating maps in Figure S3, resulting in the map in Figure 3b. Figure S7: relationship between the hyperuniformity coefficient α (the scaling coefficient of σ­(*N*(*R*))^2^) and the gap sizes for the diamond structure at varying volume fractions of high dielectric medium ϕ.(PDF)
