# Supplementary material for: Data-Driven Design Rules for Three-Dimensional Photonic Crystals
Source: ACS Omega. 2026 Mar 10;11(11):18400–7. doi: 10.1021/acsomega.6c00256 (PMC13019178; doi:10.1021/acsomega.6c00256)
Supplement: Supplementary file 1 [file ao6c00256_si_001.pdf]

Supplementary Information: Data-Driven Design Rules for Three-Dimensional Photonic Crystals

Rose K. Cersonsky*
Department of Chemical and Biological Engineering, University of Wisconsin - Madison, Madison, WI, USA
Department of Materials Science and Engineering, University of Wisconsin - Madison, Madison, WI, USA and
Data Science Institute, University of Wisconsin - Madison, Madison, WI, USA

Saswat K. Nayak
Department of Chemical and Biological Engineering, University of Wisconsin - Madison, Madison, WI, USA

Seungmin H. Lee
Department of Chemical and Biological Engineering, University of Wisconsin - Madison, Madison, WI, USA and
Department of Chemical and Biological Engineering, Notre Dame University, South Bend, IN, USA

I. ADDITIONAL SUPPORTING FIGURES TO THE MAIN TEXT

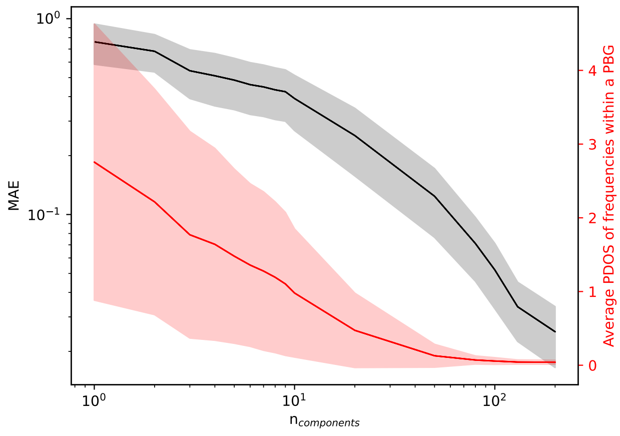

FIG. S1: Information loss in reducing photonic densities of state (PDOS) to their principal components. Black line denotes the mean absolute error (MAE) in PDOS construction across all structures. Red line denotes the mean accuracy across all structures in identifying zero-mode frequencies. Shaded regions denote one standard deviation.

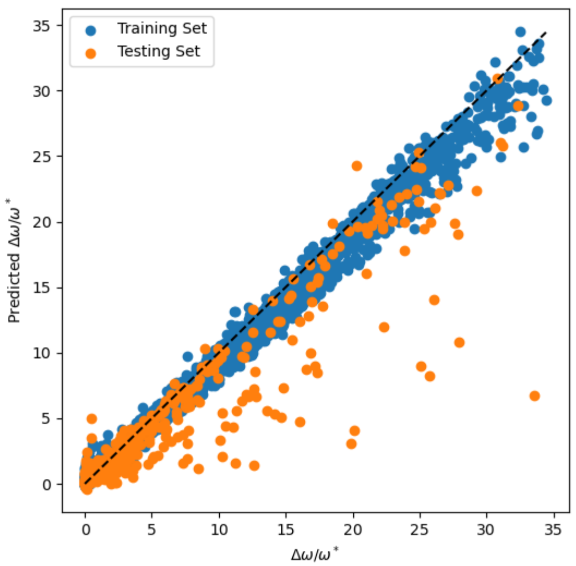

FIG. S2: Regularized kernel ridge regression relating PDOS to gap size.

* rose.cersonsky@wisc.edu

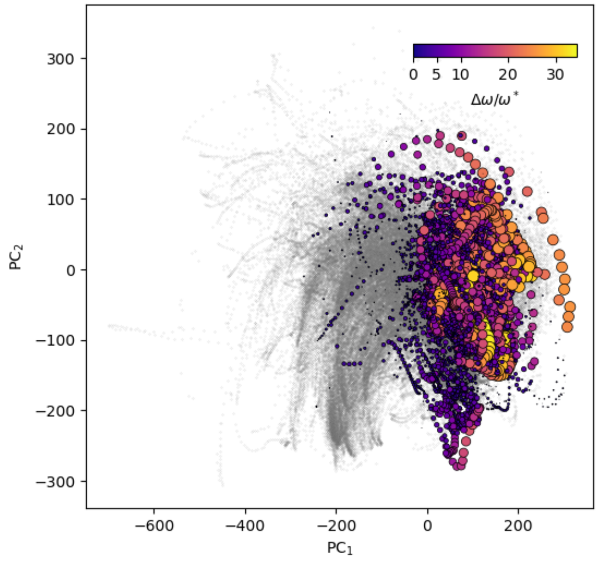

FIG. S3: Principal components analysis of the photonic densities of state, highlighting similarities in high-mode frequencies.

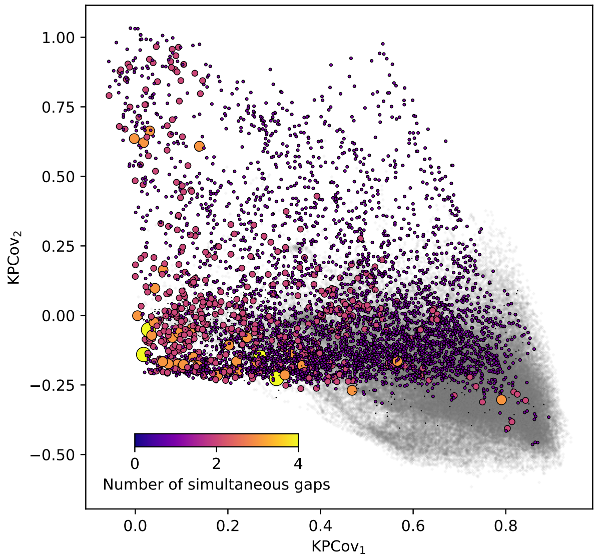

FIG. S4: Fig. 3b, colored by number of band gaps.

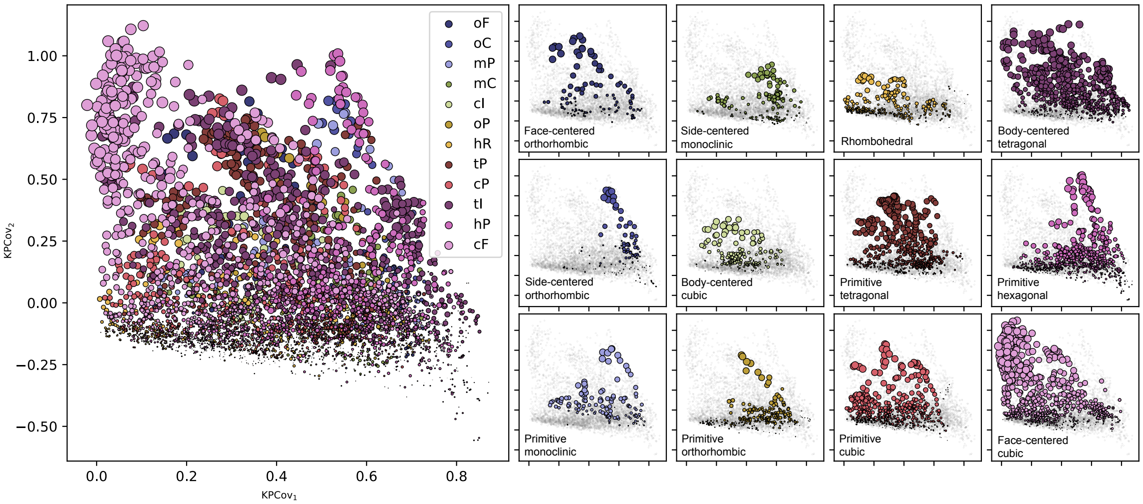

FIG. S5: Fig. 3b, colored by Bravais Lattice.

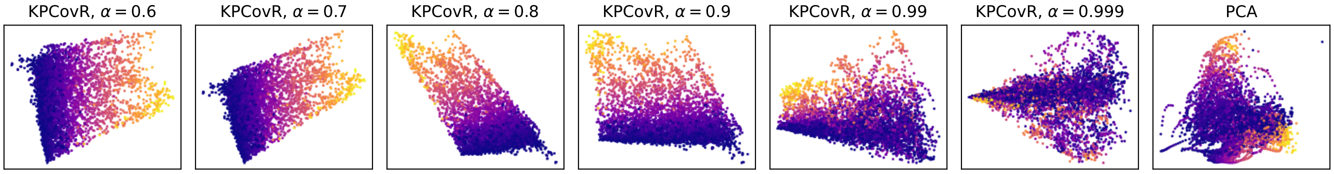

FIG. S6: Effect of rotating maps in Fig. S1, resulting in map in Fig. 3b.

II. UNSUCCESSFUL ATTEMPTS AT STRUCTURE-PROPERTY RELATIONS

Over the past several years, we have attempted to identify crystallographic descriptors and characteristics that could be used to identify and understand photonic band gap crystals as well as to construct surrogate models for band structure calculations. Below we summarize the different attempts and the conceptual or programmatic limitations to each endeavor. Many people have contributed to these trials and discussions over the years, the work below is largely attributed to Seungmin (Henry) Lee and Saswat K. Nayak.

A. Prediction of the presence of photonic band gaps based on crystallographic one-hot encodings

We first aimed to identify whether crystallographic parameters could be used to predict the presence of a photonic band gap across any value of r. This constituted a dataset of roughly 1,200 crystal structures, and binary labels corresponding to whether or not there was one value of r that resulted in a band gap greater than 1%, either in the direct or inverse structure. Crystals were represented with a 130-length vector corresponding to local and global point-group symmetries, with '1' indicating that the symmetry was present in this crystal and '0' indicating that it was not. Seungmin Lee performed trials using a 90/10 train/test split on this data and queried the performance of several appropriate classification algorithms. This yielded the results:

Model Name	Precision	Recall	Accuracy	F1 Score
Linear SVC	0.561	0.8519	0.8333	0.6765
6th Degree Polynomial SVC	0.7	0.5185	0.8561	0.5957
Gaussian Naive Bayes	0.3151	0.8519	0.5909	0.46
Complement Naive Bayes	0.5	0.6667	0.7955	0.5714
Linear Regression	0.6667	0.2963	0.8258	0.4103
Random Forest	0.6842	0.4815	0.8485	0.5652

TABLE I: Performance metrics of different supervised models.

While Random Forest models showed the highest accuracy, this was largely due to imbalanced classes – the vast majority of trials resulted in a 20% false negative rate, considered subpar accuracy in materials informatics.

B. Topological descriptors

Next, we (namely, Saswat K. Nayak) aimed to featurize each instance of the crystal structure based on its topological Betti curve, in order to determine a scale-covariant representation. Topological descriptors encode the topological invariants of a system, which encode properties that will not change under continuous deformation. One such topological invariant is the Chern number, a measure of the topological character of the electromagnetic modes, which has heavily been used to describe topological phase transitions and edge states in photonic materials[1–4]. The Chern number has been a useful analytical tool for photonic band structures; however, as it requires computation of the band structure itself, it is not an appropriate choice for representation in a predictive or correlative task.

The k-th Betti number β_k corresponds to the number of k-dimensional holes in the structure at a given particle radius R. Thus, β_0 is the number of connected components in the system, β_1 is the number of loops or cycles in the system, and β_2 is the number of voids created within the system. While the Betti numbers can be used as an instantiative descriptor, they can also be combined into a general descriptor known as the *Euler characteristic*, which is defined as

$$\chi = \sum_{i=0}^{\infty} (-1)^i \beta_i.$$

However, we ran into mathematical instabilities in applying these topological descriptors to the comparison and analyses of different crystallographic structures. When we are dealing with strictly periodic systems, some Betti numbers can asymptote to infinity[5]. For example, for a simple cubic system with lattice sites at the corners of the cube, β_1= 8 for just one unit cell but it can go to infinity as the number of unit cells increases. However, if all the eight points are connected to one another, β_0 always remains 1 regardless of the lattice size. The same arguments can be extended to β_1 and β_2. We aimed to ameliorate this problem by computing the topology of crystalline supercells; however, this causes a scaling in β_1 and β_2 for which normalization is non-trivial.

C. Hyperuniformity

Hyperuniformity is a measure of the distribution of matter across a system, and is often used to design and quantify disordered photonic materials. The hyperuniformity of a system is defined by:

$$\sigma(N(R))^2 = \langle N^2(R) \rangle - \langle N(R) \rangle^2 \tag{1}$$

where $\sigma(N(R))^2$ is the variance in the number of particles N as a function of search radius R. A system is said to be hyperuniform if the $\sigma(N(R))^2$ grows more slowly than the volume of the sphere defined by R in the limit of infinite R. This definition of hyperuniformity is directly correlated to the Fourier transform of the particle correlations. While a useful construct for disordered photonics, *all* ordered crystals are considered hyperuniform, as their infinite periodicity leads to uniform distributions. To test whether the scaling coefficients of hyperuniformity relate to the band gap size, we compute the scaling coefficients (the slope of the best-fit line for $\log(\sigma(N(R))^2)$ versus $\log(R)$, subtracting the dimensionality, 3) for the diamond structure and compare them to gap sizes:

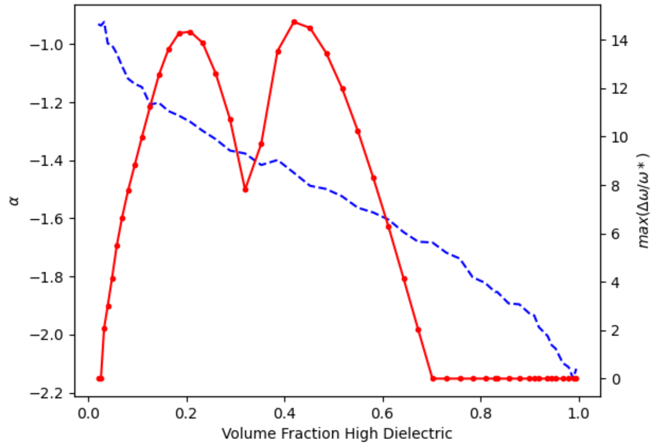

FIG. S7: Relationship between the hyperuniformity coefficient α (the scaling coefficient of $\sigma(N(R))^2$) and the gap sizes for the diamond structure at varying volume fraction of high dielectric medium ϕ.

[1] M. G. Silveirinha, Topological classification of Chern-type insulators with the photonic Green function, Physical Review B **97**, 115146 (2018), arXiv:1801.09908 [physics].

[2] C. Devescovi, M. García-Díez, I. Robredo, M. Blanco de Paz, J. Lasa-Alonso, B. Bradlyn, J. L. Mañes, M. G. Vergniory, and A. García-Etxarri, Cubic 3D Chern photonic insulators with orientable large Chern vectors, Nature Communications **12**, 7330 (2021).

[3] F. R. Prudêncio and M. G. Silveirinha, First principles calculation of topological invariants of non-Hermitian photonic crystals, Communications Physics **3**, 1 (2020).

[4] M. G. Silveirinha, Proof of the Bulk-Edge Correspondence through a Link between Topological Photonics and Fluctuation-Electrodynamics, Physical Review X **9**, 011037 (2019).

[5] H. Edelsbrunner, T. Heiss, V. Kurlin, P. Smith, and M. Wintraecken, The Density Fingerprint of a Periodic Point Set, Leibniz International Proceedings in Informatics (LIPIcs) , 16 pages, 3228454 bytes (2021), arXiv:2104.11046 [cs, math].
